# Evaluation of Tension and Deformation in a Mandibular Toronto Bridge Anchored on Three Fixtures Using Different Framework Materials, Abutment Systems, and Loading Conditions: A FEM Analysis

**DOI:** 10.1055/s-0042-1758785

**Published:** 2023-01-25

**Authors:** Francesco Grande, Pozzan Mario Cesare, Edoardo Mochi Zamperoli, Camilla Martina Gianoli, Francesco Mollica, Santo Catapano

**Affiliations:** 1 Department of Mechanical and Aerospace Engineering, Politecnico di Torino, Turin, Italy; 2 Department of Prosthodontics, University of Ferrara, Ferrara, Italy; 3 CIR Dental School Department of Surgical Sciences, University of Turin, Torino, Italy; 4 Department of Engineering, University of Ferrara, Ferrara, Italy

**Keywords:** All-on-Three, implant, prosthesis, FEM analysis, OT-Bridge, MUA

## Abstract

**Objective** The aim of this study was to investigate by finite element method analysis the behaviour of a three-implant mandible Toronto framework made by three different materials, with two abutment systems and two loading conditions.

**Materials and Methods** Three implants were virtually inserted in a mandible model in positions 3.6, 4.1, and 4.6. Three prosthetic framework bars with the same design and dimension (4.8 × 5.5 mm) were projected. The variables introduced in the computer model were the framework materials (glass fiber reinforced resin, Co-Cr, TiAl6V4), the abutment systems (Multi-Unit-Abutment [MUA]/OT-Bridge), and the loading conditions (500 N vertical load on all the framework area and 400 N on a 7-mm distal cantilever). The computer was programmed with physical properties of the materials as derived from the literature. Maximum tension and deformation values for each variable were registered at framework, screws, and abutment level and then compared.

**Results** Metal frameworks Cr-Co and TiAl6V4 resulted in lower deformation than glass fiber-reinforced resin frameworks while presenting higher tension values. The OT-Bridge exhibited lower maximum tension and deformation values than the MUA system. The first loading condition reached higher tension and deformation values than the second and it resulted in more uniformly distributed load on all the framework area, especially with the OT-Bridge system.

**Conclusion** More rigid materials and OT-Bridge system decrease the deformation on the prosthetic components. Tension stresses are more uniformly distributed with glass fiber-reinforced resin, in the OT-Bridge system and avoiding cantilever loading.

## Introduction

Nowadays, one of the main indications for the rehabilitation of edentulous patients, even with extensive bone atrophy, is represented by fixed implant prosthesis. 1 2 3 Depending on the quality and quantity of residual bone, the level of atrophy, and nerves positions, several implant rehabilitations can be performed. 4 5 6 7 Due to anatomical risk, the presence of the tongue, and the low quantity of keratinized tissue, the full-arch rehabilitation of the mandible represents a challenging procedure for dental surgeons. However, the arch described by the mandible is shorter than that of the upper arch, allowing the rehabilitation of a fixed implant prosthesis with four or also three implants. The All-On-Four technique, initially proposed by Maló et al 8 is nowadays used by several clinicians. 9 10 11 Different connection systems between prosthetic framework and implant fixtures in screw-retained rehabilitation are today available. For full-arch rehabilitations, the most used is the Multi-Unit-Abutment (MUA) system, available in a variety of size and angulations to achieve a passive prosthetic fit even in case of implant disparallelism. 7 12 13 14

A valid alternative to MUA is represented by the OT-Bridge system (Rhein 83 S.R.L., Bologna, Italy), recently introduced in the market and also investigated in scientific studies. 15 16 17 It consists of a low-profile attachment (OT-Equator), a peculiar cylindrical abutment with an “Extragrade” region that houses an interchangeable acetal ring (Seeger ring), allowing a retention with the OT-Equator even in the absence of the tightening screw. 16 The OT-Equator attachment has been initially ideated and used only for overdenture, demonstrating a long-term surveillance on these types of rehabilitation. 18 19 20 21 Its use in combination with the Extragrade abutment allows the realization of fixed prosthesis also on tilted implants with several degree of divergence. Some studies comparing MUA and OT-Bridge systems has been conducted. 15 16 In all of these, an All-On-Four model was used to evaluate differences between the two systems in terms of preload loss. The OT-Bridge system was tested also without one or two screws to understand the possible clinical implication of avoiding the screw insertion. No significant loss of screw tightening force was detected in comparison to the MUA system after approximately 1-year of cyclic loadings and the OT-Bridge system performed well even in absence of one or two prosthetic screws. In a multicenter study with a 1-year evaluation period, the OT-Bridge system showed successful results when used to support maxillary fixed dental prosthesis delivered on four to six implants. 21

In the past few years, another prosthetic alternative to All-On-Four has emerged: the All-On-Three technique. 22 23 24 25 This type of rehabilitation, specifically of the mandible, is gaining ground because of its reduced invasiveness, safety, and predictable results in the short-medium term. 8 25 26 However, the connection system, the loading conditions, and the composition of the framework could play an important role in preventing or favoring mechanical complications such as fractures, deformation of the frameworks, chipping, and wear of the ceramic coating. 27 Then, testing different framework materials and abutment system under different loading conditions is necessary to understand the behaviour of this possible implant-prosthetic rehabilitation.

To the best of our knowledge, there are no studies in literature comparing different framework materials and loading conditions with MUA and OT-Bridge system in an All-On-Three. Therefore, the aim of this study was to compare the behavior of dimensionally equal All-On-Three frameworks by finite element method (FEM) analysis in two loading conditions using two different abutment systems.

## Materials and Methods

An epoxy resin model of the mandible was initially scanned by a high precision lab scanner (Optical RevEng, Open technologies S.R.L., Brescia, Italy) to obtain an STL file of the model. The mandibular geometry was simplified as a rectangular circular object by progressively reducing the number of meshes, using a three-dimensional (3D) mesh processing open-source software program (MeshLab, ISTI, Pisa, Italy).

Three implants (Nobel Parallel, Nobel Biocare, Kloten, Switzerland) were virtually inserted in the mandible, parallel to each other and perpendicular to the occlusal plane: one implant (3.75 × 10 mm) was placed near the mandibular symphysis (4.1 position) and two (4.3 × 10 mm) were positioned at the first left and right molars (3.6 and 4.6). Soft tissues were not considered.

Then, using an appropriate software program (SolidWorks 2018, Dassault Systèmes SolidWorks Corporation, Vélizy-Villacoublay, France), a prosthetic framework connecting the three implants was designed. The framework was provided of a rectangular geometry bar with a constant section of 4.8 × 5.5 mm. Three frameworks with the same design and geometry but different materials were obtained using ( Table 1 ):

**Table 1:** Mechanical characteristics of the materials used

	Materials' type	Materials	Young's modulus	Poisson's ratio
			E	v
			[GPa]	[-]
Cortical bone without cancellous	Linear isotropic elastic	Bone	12.5	0.30
MUA 618 42		Ti Gr5 ELI	105	0.34
Seeger 618 08		POM Kepital F30-03 03	3	0.44
OT-E Profile 113 35		Ti Gr5 ELI	105	0.34
Reinforced resin framework		Trilor Arch Bioloren	26	0.4
Co-Cr framework		Magnum Lucens	194	0.3
TiAl6V4 framework		TiAl6V4	105	0.34

Abbreviations: Co-Cr, cobalt-chromium; MUA, Multi-Unit-Abutment.

1. Glass fiber-reinforced resin (Trilor Arch, Bioloren S.R.L., Saronno, Italy).
2. Cobalt-chromium (Co-Cr) (Magnum Lucens, Mesa Italia S.R.L., Travagliato, Italy).
3. Titanium alloy (Ti6Al4V, Arcam AB, Mölndal, Sweden).

For each material, two frameworks were created: one with the MUA system and the other with the OT-Bridge system. As a result, the following six models were virtually generated and tested:

1. Model with glass fiber-reinforced resin framework bar and the MUA system (M1).
2. Model with Co-Cr framework bar and the MUA system (M2).
3. Model with titanium alloy framework bar and the MUA system (M3).
4. Model with glass fiber-reinforced resin framework bar and the OT-Bridge system (M4).
5. Model with Co-Cr framework bar and the OT-Bridge system (M5).
6. Model with titanium alloy framework bar and the OT-Bridge system (M6).

**Fig. 1:**
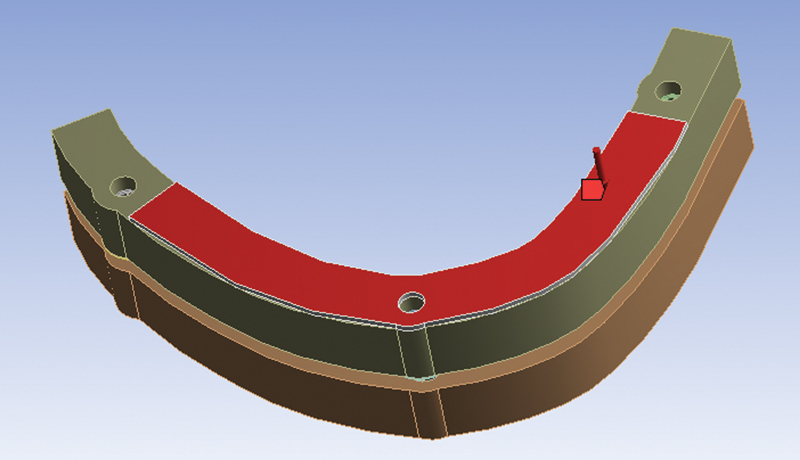
First loading condition: application of a 500 N perpendicular load on the framework area between implants in 4.1 and 3.6 positions.

**Fig. 2:**
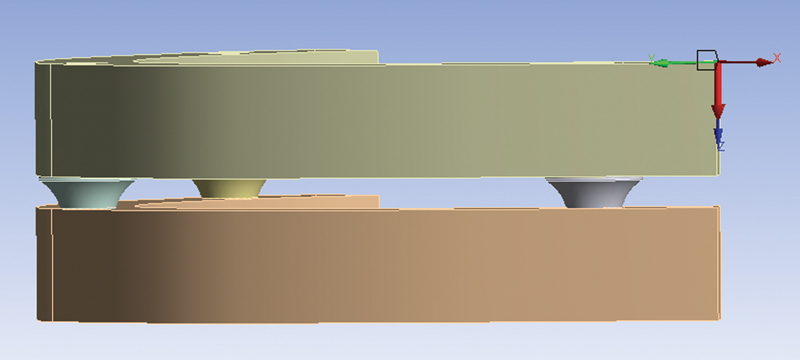
Second loading condition: application of a 400 N perpendicular load on a 7-mm distal cantilever to 4.6 position.

A FEM analysis for all the frameworks, using the ANSYS software (Ansys, Inc., Canonsburg, Pennsylvania, United States), was carried out. A 3D linear static parametric simulation was developed, considering the ratio (stress and strain) between bone and prosthetic components, and implants and OT-Equator attachments. Two different loading conditions were applied for each FEM:

- Perpendicular load of 500 N applied on the framework area between implants 4.1 and 3.6 (first loading condition) ( Fig.1 ).
- Perpendicular load of 400 N applied on a 7-mm distal cantilever to 4.6, to generate a bending moment of the prosthetic framework (second loading condition) ( Fig.2 ).

Both in the first and second loading conditions, the maximum values were recorded in terms of tension (MPa) and deformation (mm) in the axial direction at framework, screw, and abutment level. The mechanical behavior of the different frameworks was compared as the connection/abutment system changes.

The study was carried out with the hypotheses that linear elastic and isotropic materials, subjected to a tension, undergo an elastic deformation proportional to the tension itself according to a proportional factor, the Young's modulus. In the program fixed conditions were established in terms of linear elastic and isotropic material deformation. For the elasticity, the materials were subjected to a proportional deformation according to the Young's modulus of the material. It was also fixed that the material had the same mechanical and thermal properties in all directions (isotropy).

## Results

Results are summarized in Tables 2 3 4 5 . The incidence of each variable on tension and deformation stresses at framework, screws, and abutment level was analyzed by matching the different results in several ways. Bar graphs of each comparison are attached as Supplementary File .

**Table 2:** Tensions values ([Mpa] σeq,vm) expressed in the first loading condition for each abutment system and framework material at framework, screw, and abutment level

	Abt system	Framework		Screws		Abt	
		Value (max)	Area	Value (max)	Area	Value (max)	Area
Resin	MUA (M1)	800	3.6, 4.1, 4.6	500	3.6 thread	650	3.6, 4.6 Cone bone seat
	OTP (M4)	200	3.6, 4.6	300	3.6, 4.6 thread	650	3.6, 4.6 Ot_eq and abt
Cr-Co	MUA (M2)	800	3.6, 4.1, 4.6	650	3.6 thread	680	3.6, 4.6 Cone bone seat
	OTP (M5)	650	3.6, 4.6	400	3.6, 4.6 thread	500	3.6, 4.6 Ot_eq and abt
TiAl6V4	MUA (M3)	800	3.6, 4.1, 4.6	650	3.6 thread	680	3.6, 4.6 Cone bone seat
	OTP (M6)	500	(3.6, 4.6	400	3.6, 4.6 thread and head	600	3.6, 4.6 Ot_eq and abt

Abbreviations: Abt, abutment; Cr-Co, chromium-cobalt; MUA, Multi-Unit-Abutment.

**Table 3:** Deformation values (mm) in the axial direction ( *z* -axis) expressed in the first loading condition for each abutment system and framework material at framework, screw, and abutment level

	Abt system	Framework		Screws		Abt	
		Value (max)	Area	Value (max)	Area	Value (max)	Area
Resin	MUA (M1)	1.05	4.1	0.98	4.1	1.04	4.1
	OTP (M4)	0.9	4.1	0.88	4.1	0.92	4.1
Cr-Co	MUA (M2)	0.97	4.1	0.91	4.1	0.97	4.1
	OTP (M5)	0.88	4.1	0.83	4.1	0.87	4.1
TiAl6V4	MUA (M3)	0.99	4.1	0.93	4.1	0.99	4.1
	OTP (M6)	0.89	4.1	0.85	4.1	0.88	4.1

Abbreviations: Abt, abutment; Cr-Co, chromium-cobalt; MUA, Multi-Unit-Abutment.

**Table 4:** Tensions values ([Mpa] σeq,vm) expressed in the second loading condition for each abutment system and framework material at framework, screw, and abutment level

	Abt system	Framework		Screws		Abt	
		Value (max)	Area	Value (max)	Area	Value (max)	Area
Resin	MUA (M1)	300	3.6	400	3.6 thread	650	3.6 Cone bone seat
	OTP (M4)	200	3.6	200	3.6 thread	650	3.6 Ot_eq
Cr-Co	MUA (M2)	800	3.6	400	3.6 start thread	650	3.6, 4.6 Cone bone seat
	OTP (M5)	300	3.6	170	3.6, 4.6 thread	600	3.6 Ot_eq
TiAl6V4	MUA (M3)	800	3.6	400	3.6 start thread	450	3.6 Cone bone seat
	OTP (M6)	300	3.6	170	3.6 thread		3.6 Ot_eq

Abbreviations: Abt, abutment; Cr-Co, chromium-cobalt; MUA, Multi-Unit-Abutment.

**Table 5:** Deformation values (mm) in the axial direction ( *z* -axis) expressed in the first loading condition for each abutment system and framework material at framework, screw, and abutment level

	Abt system	Framework		Screws		Abt	
		Value (max)	Area	Value (max)	Area	Value (max)	Area
Resin	MUA (M1)	0.21	4.1	0.19	4.1	0.2	4.1
	OTP (M4)	0.18	4.1	0.16	4.1	0.17	4.1
Cr-Co	MUA (M2)	0.19	4.1	0.18	4.1	0.19	4.1
	OTP (M5)	0.17	4.1	0.16	4.1	0.17	4.1
TiAl6V4	MUA (M3)	0.2	4.1	0.18	4.1	0.2	4.1
	OTP (M6)	0.17	4.1	0.16	4.1	0.17	4.1

Abbreviations: Abt, abutment; Cr-Co, chromium-cobalt; MUA, Multi-Unit-Abutment.

### Materials

Regarding materials in the first loading condition, glass fiber-reinforced resin with Co-Cr and titanium decreased, respectively, of 70 and 40% the maximum stress tension of the framework using OT-Bridge (M4, M5, M6). When the distal cantilever is loaded (second loading condition), the same material had up to 63% less of maximum tension values compared to Co-Cr and titanium material in the MUA system (M1, M2, M3). Screws and abutment tension was not so affected by framework material.

Instead, deformation occurred for all the prosthetic components in all models in the range of 0.16 to 0.21 mm and resulted always higher for glass fiber-reinforced resin than metal frameworks.

### Abutment System

In most of the cases, changing the connection type from MUA to OT-Bridge reduced tension values. Notable differences were observed in M1 versus M4 (800 vs. 200 MPa) and with Co-Cr and titanium at framework level applying the second loading condition (decrease of 37.5%). Screw tensions comparing the models showed a decrease from 40% in the first loading condition to 67.5% in the second loading condition. At abutment level, changing MUA with OT-Bridge slightly decreased the tension values only with Co-Cr (M2 vs. M5) especially in the first loading condition; a raise of 25% was observed with titanium (M3 vs. M6) in the second loading condition and was the only case in which tension values increased when passing from MUA to OT-Bridge. OT-Bridge showed a larger distribution of tension stresses across the framework, reducing their intensification in small areas for both the loading conditions.

Changing abutment system slightly influenced the deformation level of the different prosthetic components. A mild decrease of deformation values was observed when using OT-Bridge instead of MUA; the maximum value of difference (0.15 mm) was reached at framework level with M1 versus M4 in the first loading condition.

### Loading Conditions

In all the models tested, the first loading condition reached higher tension stress and deformation values than the second loading condition. By passing from the first to the second loading condition, tension values decreased, in some cases, up to 62,5% at abutment (M1) and screw (M5, M6) level. Also, the deformation of the framework in axial direction ( *z* -axis) decreased by about 80% in the second loading condition for all the materials and in all the components. However, the deformation of the framework bar was less uniformly distributed when the load is applied to the distal cantilever, resulting in higher deformation near the closest abutment similarly to tension distribution. In addition, in the first loading condition the anterior area of the framework reached maximum deformation while the distal portion had the lowest; changing loading set inversed the deformed location.

## Discussion

Full-arch implant prosthesis must be planned and fabricated considering the distribution of forces on implant components. Materials and abutment system affected the survival rate of implants, prosthesis, and the onset of mechanical complications. Despite all its inherent limitations, 28 FEM analysis represents a valid tool for studying the behaviour of implant-prosthetic components with different configurations, giving initial evaluation on the feasibility of a rehabilitation. 29

The purpose of this FEM was to evaluate the mechanical behavior of a fixed prosthesis anchored on three implants using different materials for the framework bar, different connection system, and applying two different loading conditions to simulate occlusion. A mandibular model with all cortical bone and without cancellous was projected to maximize stresses at implant-prosthetic components, simulating the worst case scenario. 30 Perfect passivity between the components was assumed to avoid the appearance of internal tensions that may confound the analysis.

### Materials

Co-Cr and titanium alloy frameworks generate greater tensions that were largely distributed, involving less framework deformation. Oppositely, despite the lower tensions, the glass fiber-reinforced resin material tends to concentrate them in a smaller surface, thus exposing the bar to greater deformations. This behaviour could be explained because of the material properties and to the rigidity provided by metal alloy. On the contrary, the lower Young's modulus of resin base framework may lead to a greater absorption of the load in the area of application without any or little tension distribution.

It is important to also consider the mechanical resistance of these materials, when planning a framework, to prevent mechanical complications such as chipping, fractures, and screw loosening under masticatory loads. Because of the lower rigidity and higher shock absorbance concentration of glass fiber-reinforced resin compared to metal alloys frameworks, it is important to plan a proper framework dimension when choosing this material. In addition, it is important to correctly select the material in relation to the clinical case. A higher framework resistance could be preferable in patients with higher masticatory forces and smaller loading distribution. Furthermore, for definitive restorations with ceramic coatings, it may be recommended to select a framework material that does not expose the bar to deformations in order to reduce chipping and fracture risks. Proper framework design is necessary in relation to the specific clinical case, number and position of implants, and to the material used.

### Abutment

**Fig. 3:**
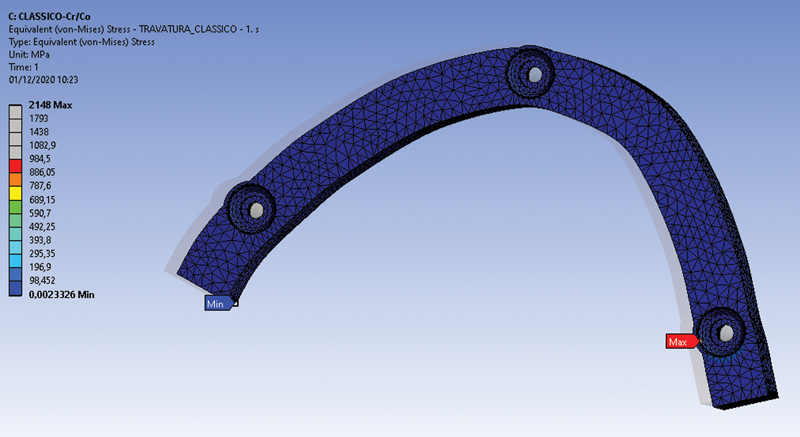
Tension distribution at framework area of M2 (cobalt-chromium framework bar and Multi-Unit-Abutment [MUA] system) in the second loading condition. Tension values are expressed by a colorimetric scale. The location of minimum and maximum values are indicated.

**Fig. 4:**
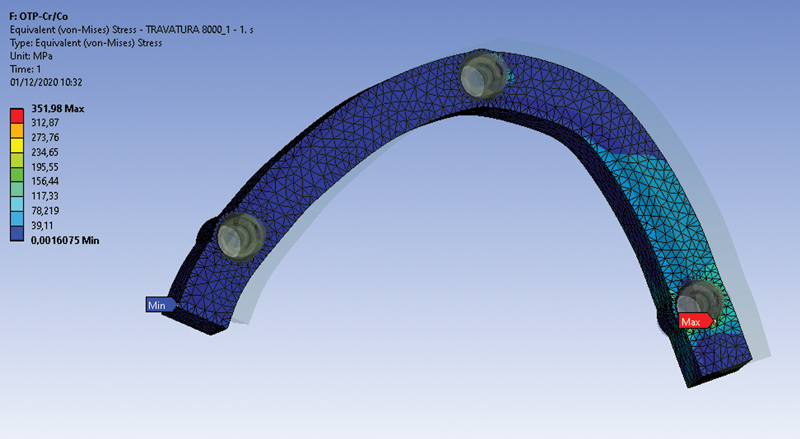
Tension distribution at framework area of M4 (glass fiber-reinforced resin framework bar and OT-Bridge system) in the second loading condition. Tension values are expressed by a colorimetric scale. The location of minimum and maximum values are indicated.

More concentrated local tensions were recorded and observed with the MUA system. Results proved that OT-Bridge distributes the acting tensions more uniformly, reducing their intensification for both the loading conditions. This could be due to the OT-Equator structural configuration which seems to collect the strength over the head of the retainer and not only in a single point 31 ( Figs. 3 and 4 ).

Another important result is that higher tensions and deformation were reached by MUA at all levels. This phenomenon could be described by the low profile and overall smaller size of the OT-Equator attachment which provides the framework of a greater dimensions and consequently greater mechanical properties. In addition, it is possible that in the OT-Bridge system, a portion of stress is transmitted to the Seeger ring which provides the interlocking connection between the implant and the prosthesis. Then, if an amount of energy is dissipated in this way, especially in case of distal cantilever loading, a reduction of the transmitted energy happens, decreasing stresses.

### Loading Conditions

In the first loading condition, the distribution of 500 N load was extended over a large area inside the framework arch determining an overall greater stress on both the connection types (MUA or OT-Bridge). Passing from the first to the second loading condition (400 N applied on a 7-mm distal cantilever), tension and deformation decreased for all the tested models. However, the deformation was much more concentrated in the region where the load is applied near the distal cantilever. This is in relation to loading application, involving a bending moment of the prosthetic framework with related tension in all the prosthetic components, especially in the bar region between the anterior and the distal implant after the first implant prosthetic connection.

Other FEM analysis in literature evaluating framework materials, abutment systems, and loading conditions can be found. Rubo et al 30 found that stress increase proportionally to the increase in cantilever length and inversely to the increase in the elastic modulus of cancellous bone. Moreover, they concluded that a stiffer framework may allow better stress distribution, which is in accordance with our study results. This relation about rigidity and stress distribution was also highlighted by other studies testing polymeric materials (polyether ether ketone and polyetherketoneketone) at FEM. 32

Regarding abutment type, rigid abutment design showed to decrease the peak stresses in the screw and the deflection of the superstructure. 33 34 In addition, in another study, the conical implant connected to a solid, internal, conical abutment furnishes lower stresses on the alveolar bone and prosthesis and greater stresses on the abutment compared to a stepped cylinder implant connected to a screw-retained, internal hexagonal abutment. 35 In our study, we focused only on the prosthetic components, but it is important to underline that the design of implant-abutment interface could affect the surrounding bone tissue. Conical implant-abutment interface decreased the stress at bone-implant interface, resulting also in a more apical shear stress transmission compared to the flat top interface. 36

About the OT-Bridge system, only one FEM was published by Cervino et al 17 ; however, they focused on the stability of OT Bridge prosthesis in an All-On-Four mandibular model, concluding that at maximum one abutment can be unscrewed to ensure an adequate stability of the system. In another FEM evaluating different overdenture attachments, 31 the locator and OT-Equator system was found to offer better stress distribution compared to the traditional universal abutment. Furthermore, OT-Equator favored a higher stress on the retainer gum with minor stress located around the peri-implant bone tissue and fixture. This favorable stress distribution of OT-Equator attachment was also recognized in our study, where it was able to dissipate stress tensions over an extended area, differently from MUA.

Concerning forces application, the literature demonstrated that cantilever loading increased stress proportionally to its length. 30 In addition, stresses clustered at the elements closest to the loading point distribution. This is in accordance with our study where tension stresses in the cantilever loading condition was spread especially in the portion of the framework near the closest abutment. Literature provided also evidence that axial and nonaxial occlusal loads influence both stress distribution on prosthetic components and on the bone remodeling phenomena. 37 38

Despite the advantages of finite element analysis in the biomedical area, 29 a virtual simulation of a clinical condition using a computer software is limited. 39 In addition, in this study, only two loading conditions in two different moments were tested with a unidirectional loading while the occlusion normally produces multidirectional forces also in simultaneous moments. The framework design and the section of the bar are also related to the clinical case, and this could affect the mechanical properties and the distribution of the loading forces. Furthermore, ceramization of the prosthetic framework may change the pattern and the magnitude of occlusion forces distribution on framework bar, screws, and abutment. Then, further investigation and studies with more samples, and possibly *in* *vivo* conditions are required to evaluate the effectiveness of the All-On-Three technique and of the OT-Bridge system.

## Conclusion

Metal alloy materials reduce the framework deformation during loadings because of the high mechanical properties. The OT-Bridge system raised the mechanical properties of the framework because of its smaller size and spread tension stresses more uniformly than MUA. Cantilever loading concentrated stress tensions and deformation in smaller areas for all the connection systems.
