# Supplementary material for: Evaluation of Tension and Deformation in a Mandibular Toronto Bridge Anchored on Three Fixtures Using Different Framework Materials, Abutment Systems, and Loading Conditions: A FEM Analysis
Source: Eur J Dent. 2023 Jan 25;17(4):1097–105. doi: 10.1055/s-0042-1758785 (PMC10756777; doi:10.1055/s-0042-1758785)
Supplement: Supplementary file 1 — Supplementary Material [file 10-1055-s-0042-1758785-s2262201.pdf]

Supplementary File

Results

Materials

Tension values ([Mpa] σeq,vm) for reinforced resin, Co-Cr, and Ti material in the first and second loading condition.

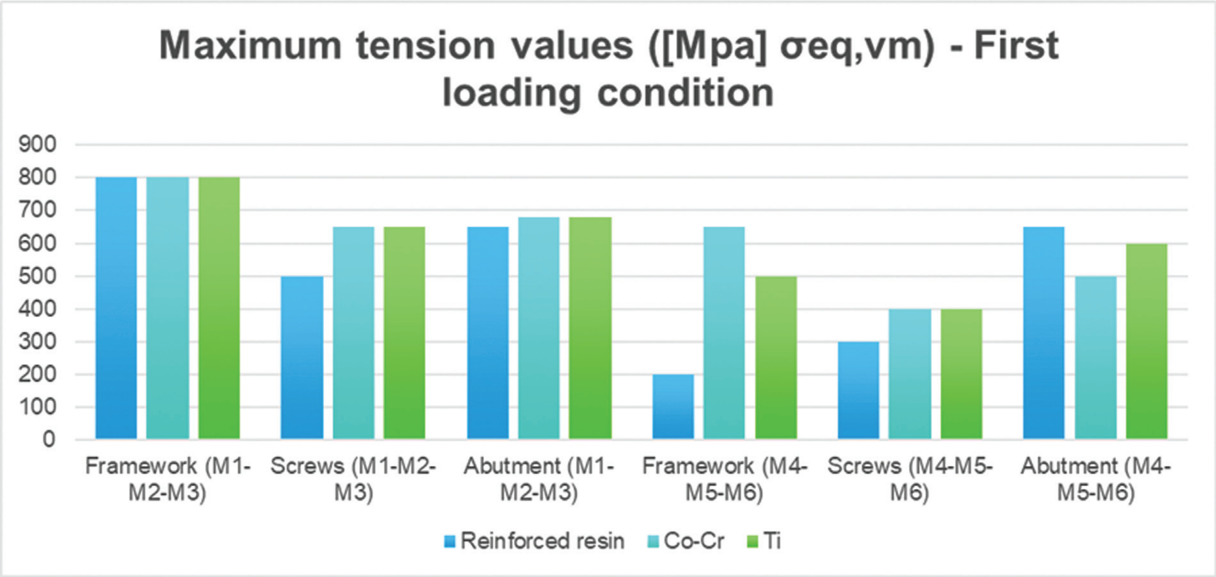

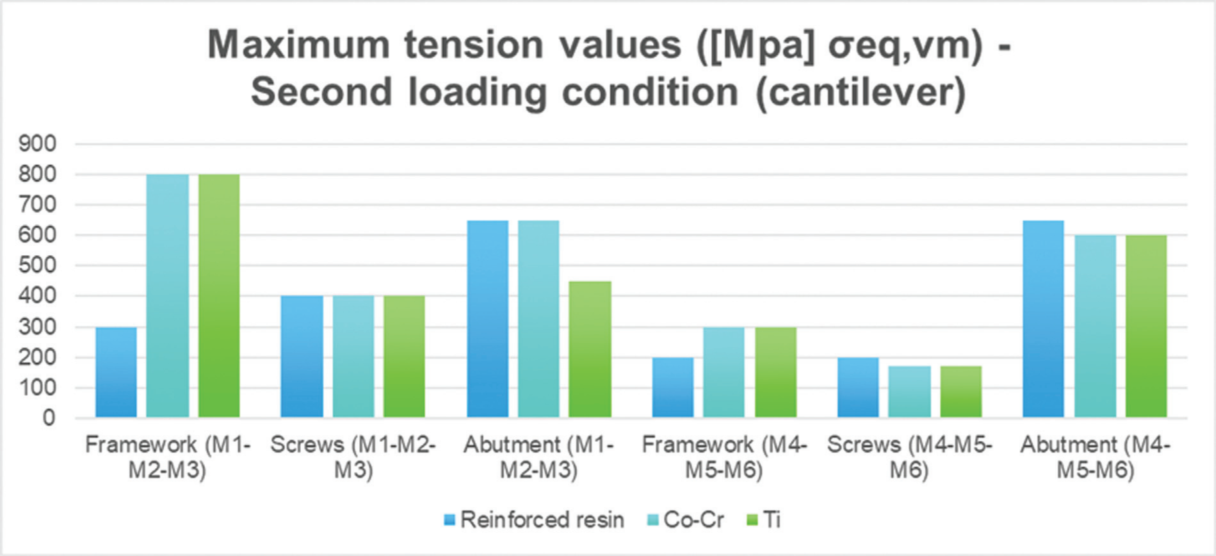

 © 2023. The Author(s).

Deformation values (mm) for reinforced resin, Co-Cr, and Ti material in the first and second loading condition.

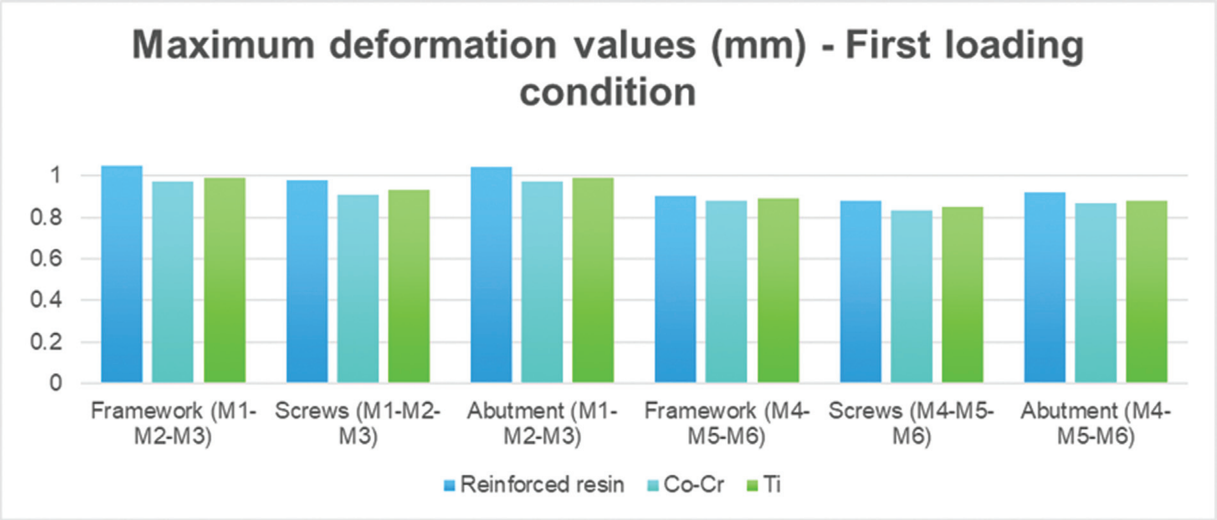

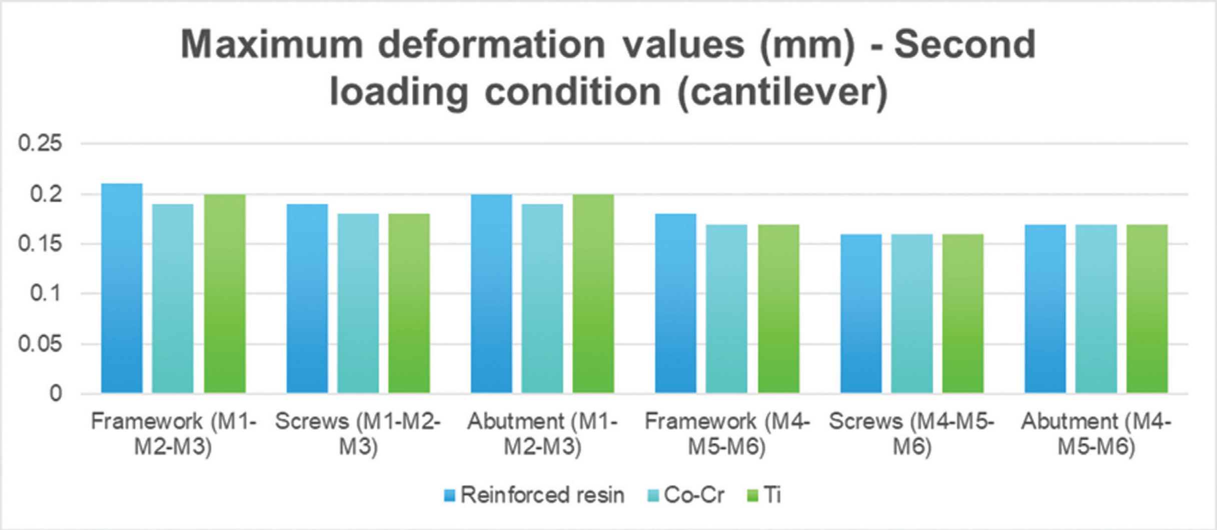

 © 2023. The Author(s).

Abutment System

Tension values ([Mpa] σeq,vm) for MUA and OT-Bridge system at framework, screws, and abutment level.

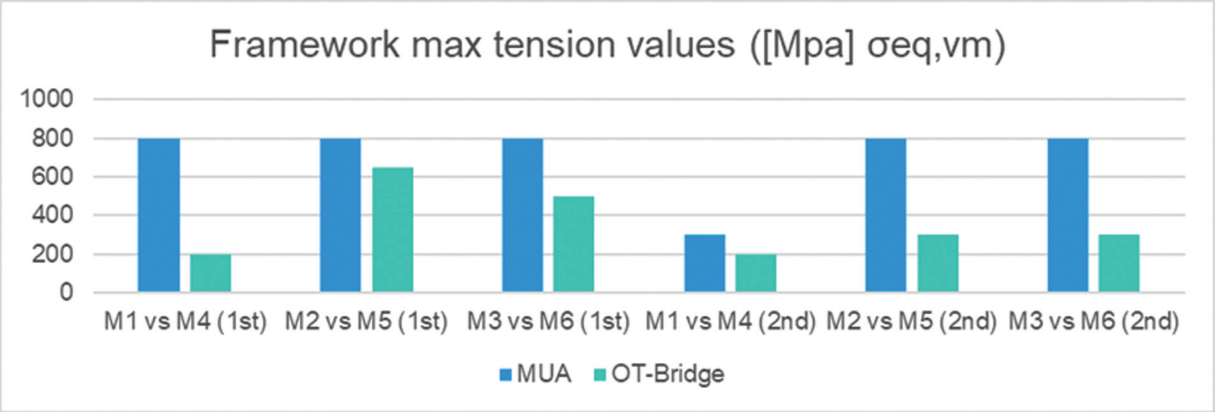

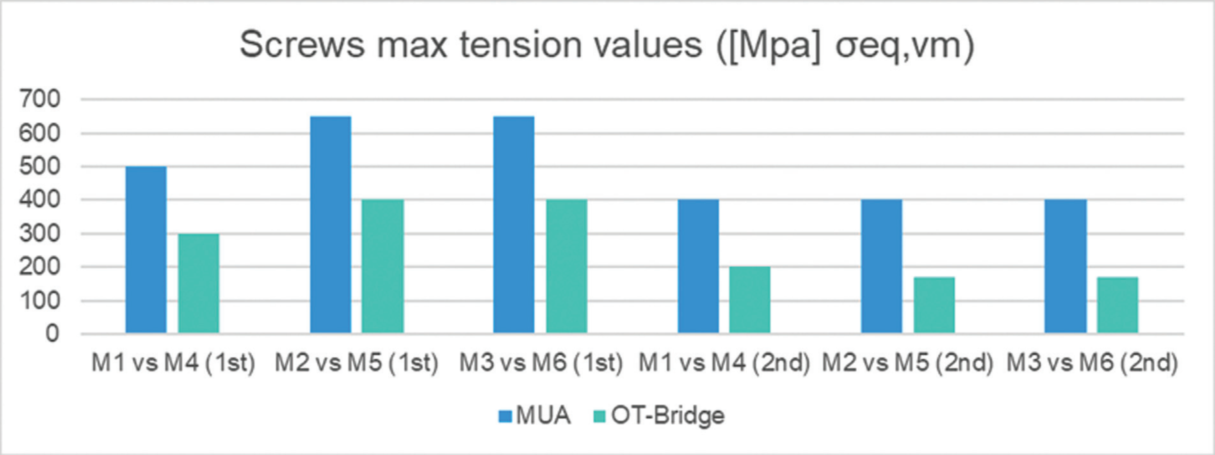

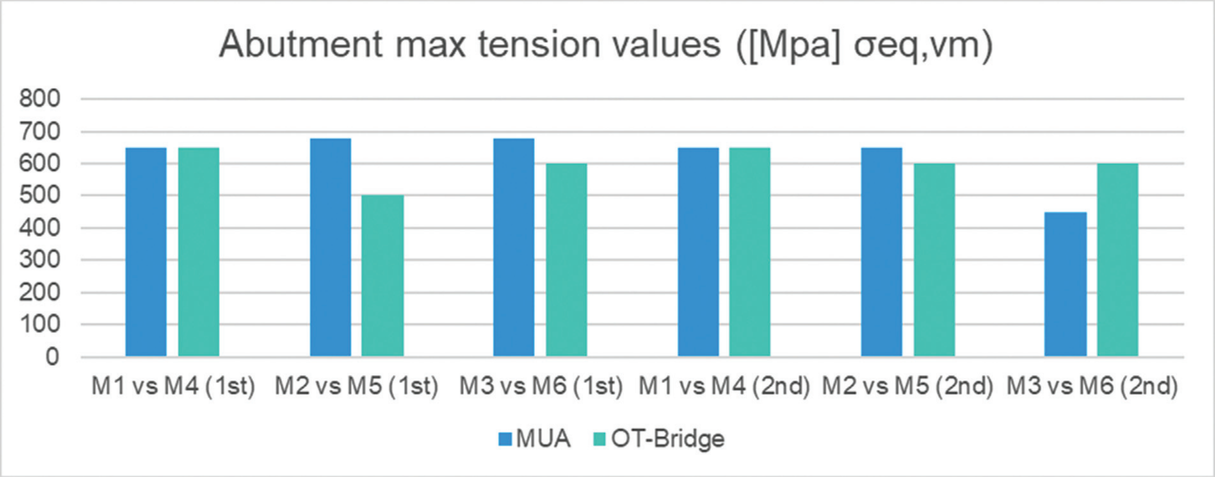

 © 2023. The Author(s).

Deformation values (mm) for MUA and OT-Bridge system at framework, screws, and abutment level.

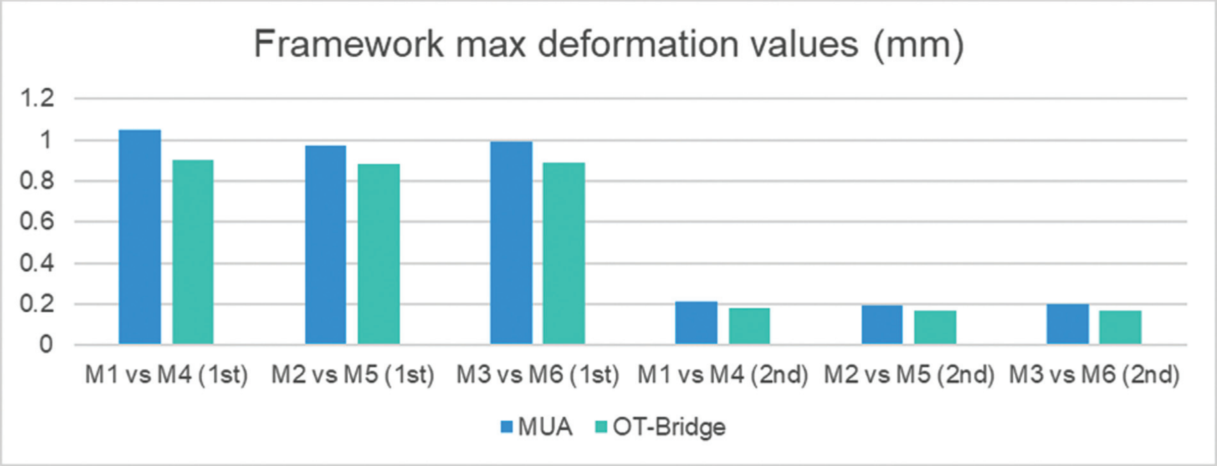

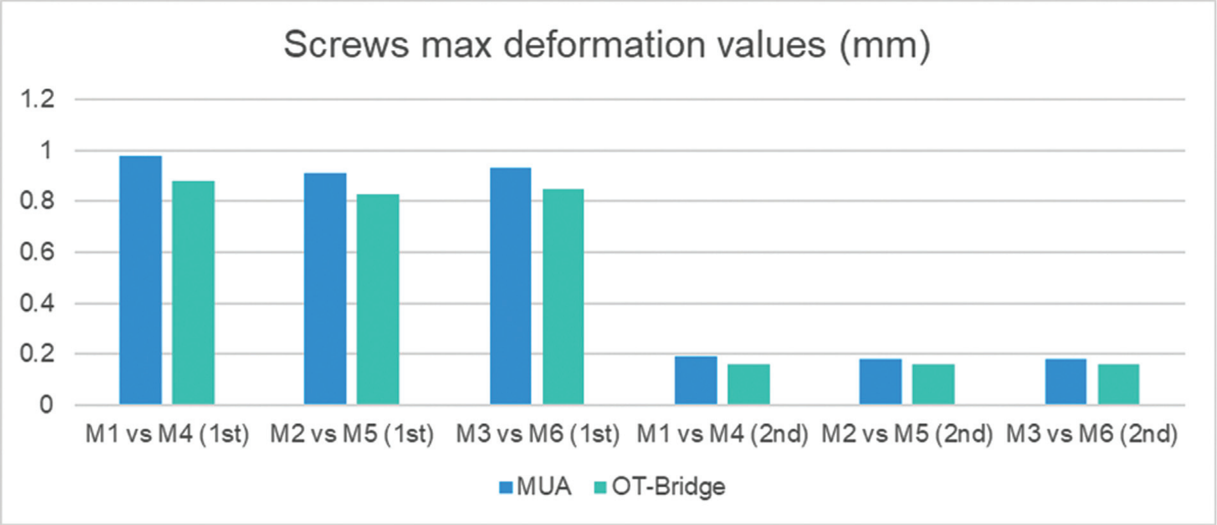

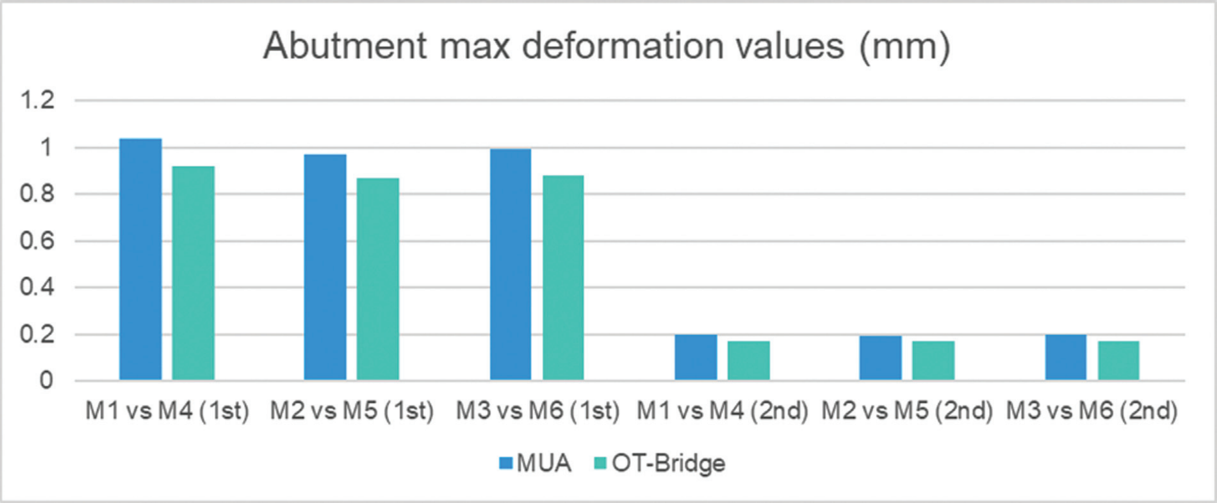

 © 2023. The Author(s).

Loading Conditions

Tension values ([Mpa] σeq,vm) for the first and second loading conditions at framework, screws, and abutment level.

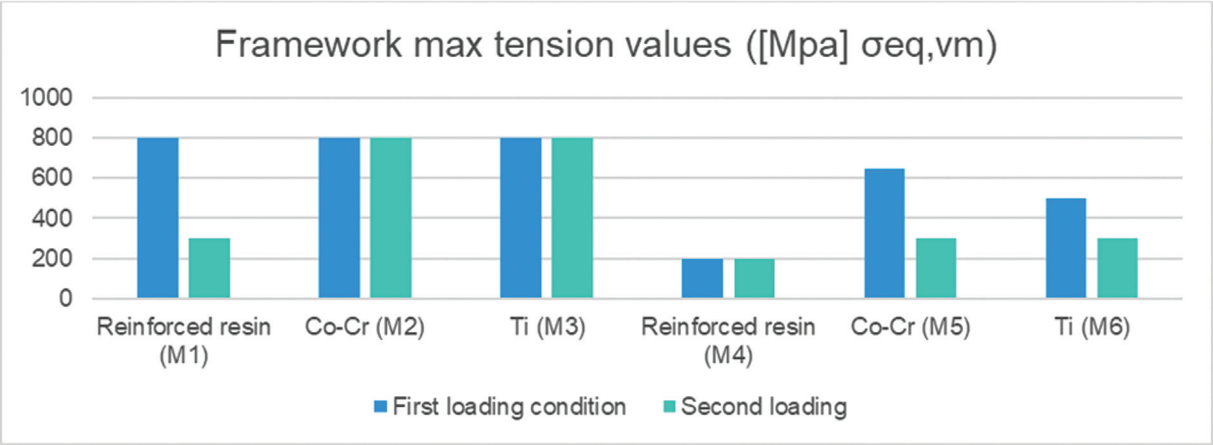

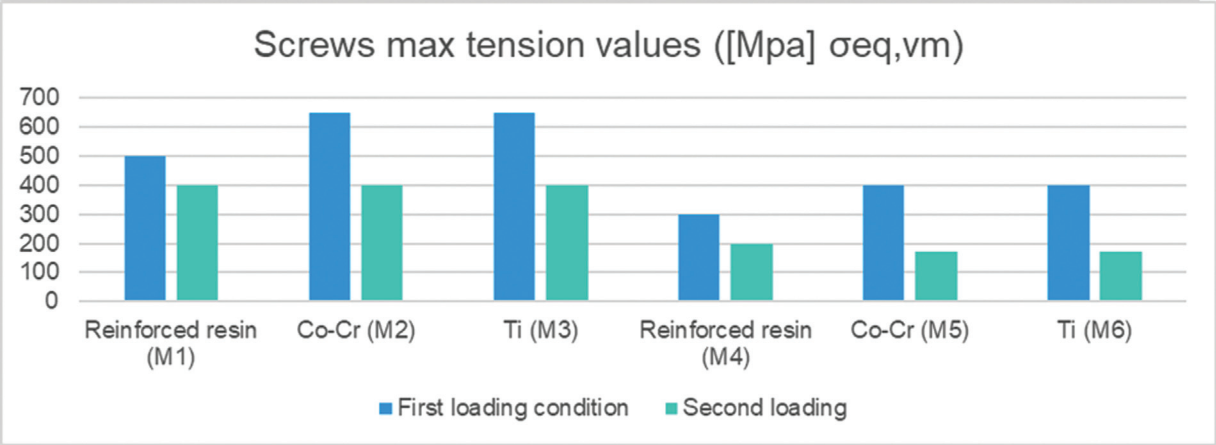

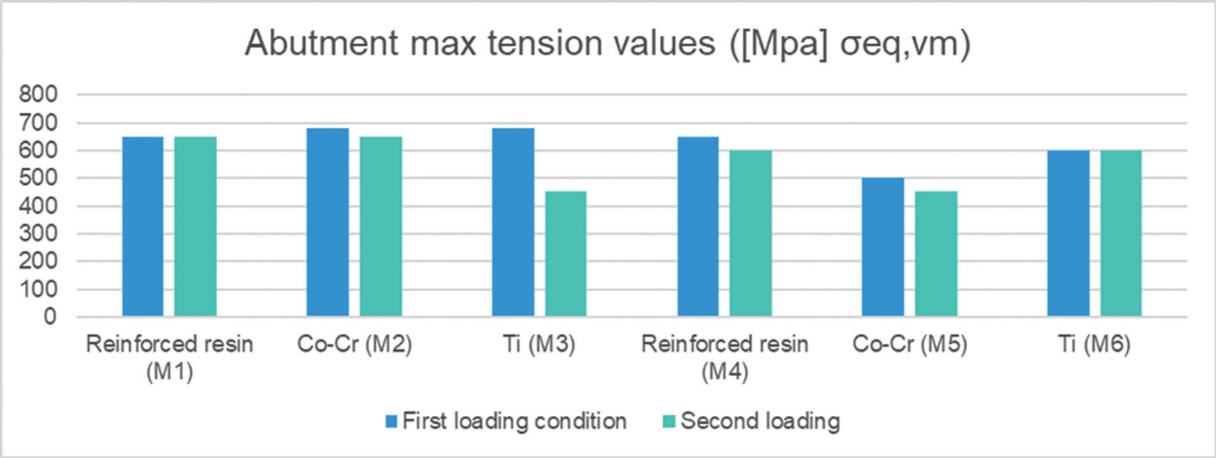

 © 2023. The Author(s).

Deformation values (mm) for the first and second loading conditions at framework, screws, and abutment level.

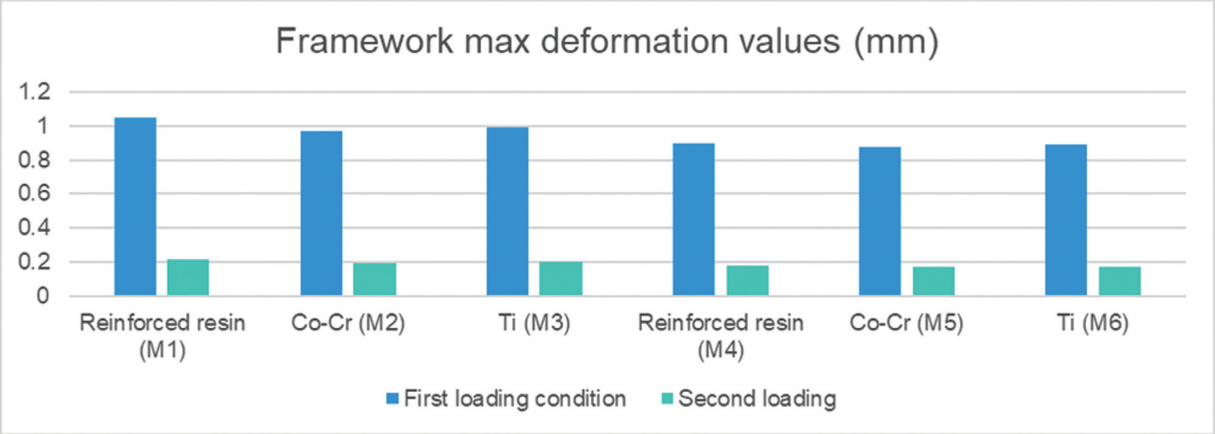

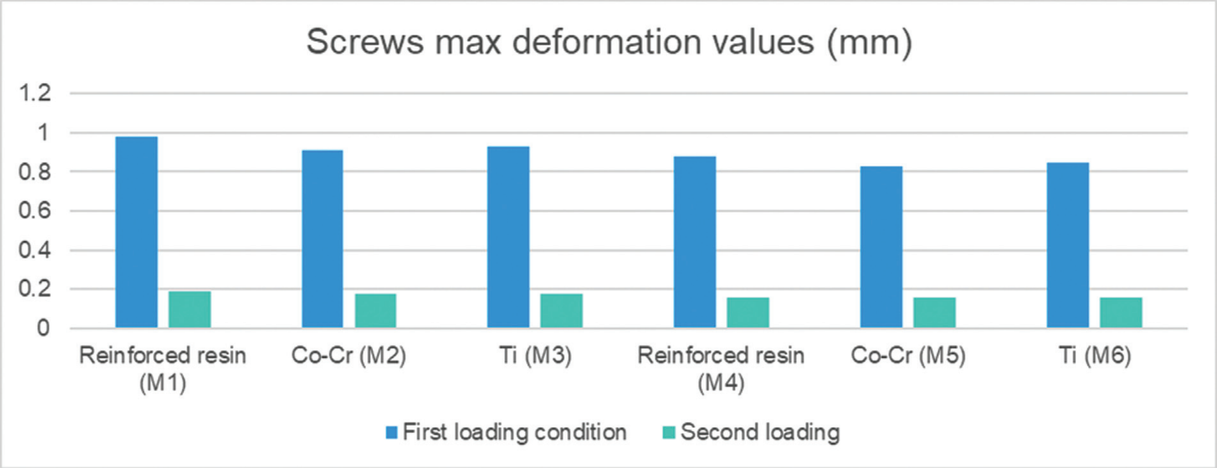

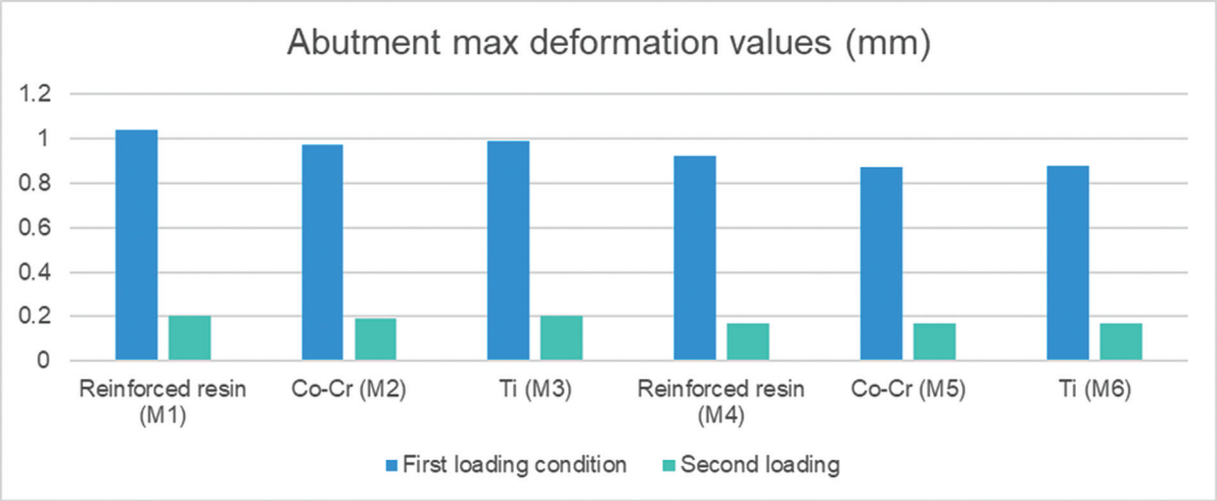

 © 2023. The Author(s).
